# Protein kinase C zeta suppresses low‐ or high‐grade colorectal cancer (CRC) phenotypes by interphase centrosome anchoring

**DOI:** 10.1002/path.5035

**Published:** 2018-03-09

**Authors:** Ravi Kiran Deevi, Arman Javadi, Jane McClements, Jekaterina Vohhodina, Kienan Savage, Maurice Bernard Loughrey, Emma Evergren, Frederick Charles Campbell

**Affiliations:** ^1^ Centre for Cancer Research and Cell Biology Queen's University of Belfast Belfast UK; ^2^ Northern Ireland Molecular Pathology Laboratory, Centre for Cancer Research and Cell Biology Queen's University Belfast and Belfast Health and Social Care Trust Belfast UK

**Keywords:** protein kinase C, centrosome, spindle apparatus, chromosomal instability, colorectal neoplasms

## Abstract

Histological grading provides prognostic stratification of colorectal cancer (CRC) by scoring heterogeneous phenotypes. Features of aggressiveness include aberrant mitotic spindle configurations, chromosomal breakage, and bizarre multicellular morphology, but pathobiology is poorly understood. Protein kinase C zeta (PKCz) controls mitotic spindle dynamics, chromosome segregation, and multicellular patterns, but its role in CRC phenotype evolution remains unclear. Here, we show that PKCz couples genome segregation to multicellular morphology through control of interphase centrosome anchoring. PKCz regulates interdependent processes that control centrosome positioning. Among these, interaction between the cytoskeletal linker protein ezrin and its binding partner NHERF1 promotes the formation of a localized cue for anchoring interphase centrosomes to the cell cortex. Perturbation of these phenomena induced different outcomes in cells with single or extra centrosomes. Defective anchoring of a single centrosome promoted bipolar spindle misorientation, multi‐lumen formation, and aberrant epithelial stratification. Collectively, these disturbances induce cribriform multicellular morphology that is typical of some categories of low‐grade CRC. By contrast, defective anchoring of extra centrosomes promoted multipolar spindle formation, chromosomal instability (CIN), disruption of glandular morphology, and cell outgrowth across the extracellular matrix interface characteristic of aggressive, high‐grade CRC. Because PKCz enhances apical NHERF1 intensity in 3D epithelial cultures, we used an immunohistochemical (IHC) assay of apical NHERF1 intensity as an indirect readout of PKCz activity in translational studies. We show that apical NHERF1 IHC intensity is inversely associated with multipolar spindle frequency and high‐grade morphology in formalin‐fixed human CRC samples. To conclude, defective PKCz control of interphase centrosome anchoring may underlie distinct categories of mitotic slippage that shape the development of low‐ or high‐grade CRC phenotypes. © 2018 The Authors. *The Journal of Pathology* published by John Wiley & Sons Ltd on behalf of Pathological Society of Great Britain and Ireland.

## Introduction

Colorectal cancer (CRC) may represent the major cancer challenge of the 21st century because it is the third most lethal global malignancy and its incidence is expected to increase by 60% over the next two decades 1. Growth of CRC ranges from indolent to highly aggressive 2. Prognostic stratification is aided by histological grading 3, 4 but aggressive CRC is characterized by chromosome segregation error 5 as well as high‐grade morphology 3, 4. Chromosome partitioning is coupled to multicellular morphology by mitotic apparatus 6, 7 and relevant interplay may be dissected in organotypic culture models.

Ordered cell division maintains the epithelial barrier in the healthy colon 8. In preparation for mitosis, cells copy their genome and remodel their internal architecture to enable mitotic spindle assembly 9. In Caco‐2 CRC cells, redistribution of the cytoskeletal linker protein ezrin to form a cap‐like accumulation at one pole of the cell cortex provides a cue for astral microtubule (MT) capture and stabilization of the interphase centrosome 10. Thus anchored, the centrosome normally replicates to generate one mother and one daughter 10. Centrosome anchoring to the cell cortex is necessary for separation of mother and daughter centrosomes 11, construction and orientation of the mitotic spindle 12, formation of cell shape 13, and multicellular assembly 12. Not only does the ezrin cap stabilize the normal centrosome, it also anchors and ‘clusters’ extra centrosomes during interphase 10. Centrosome amplification characterizes many human cancers 14 and may be driven by polo‐like kinase 4 (PLK4) overexpression 15. Effective clustering of extra centrosomes during interphase enables assembly of a bipolar mitotic spindle, error‐free segregation of a diploid chromosome complement 16, and normal multicellular pattern formation 10. Conversely, ineffective clustering of interphase centrosomes can activate failsafe processes that cluster extra centrosomes later in the cell cycle, during metaphase 16. However, these metaphase centrosome clustering processes invoke substantive segregation error 16.

While molecular controls of ezrin spatiotemporal dynamics remain unclear, the polarity regulator protein kinase C zeta (PKCz) phosphorylates ezrin to initiate embryonic morphogenesis 17. PKCz also controls centrosome positioning 18, orientated mitosis 19, chromosome segregation 20, and multicellular assembly 21. In this study, we dissected PKCz regulation of ezrin interactions with its known binding partner NHERF1 22 [also known as ezrin binding protein 50 (EBP50) or solute carrier family 9, sodium/hydrogen exchanger, isoform 3, regulator 1 (SLC9A3R1)], which is important for maintenance of ezrin at the cell cortex 23. We also investigated PKCz regulation of merlin, which is known to be involved in ezrin cap formation 10. We show that perturbation of ezrin cap formation alone or in combination with centrosome amplification drives the evolution of phenotypes evocative of low‐ or high‐grade CRC, in 3D organotypic culture models.

## Materials and methods

### Reagents and antibodies

All laboratory chemicals were purchased from Sigma‐Aldrich, Dorset, UK, unless otherwise stated.

### Organotypic and organoid cultures

Caco‐2, BT‐549, and U2OS cells were obtained from ATCC, Middlesex, UK. Caco‐2 cells were grown in three‐dimensional (3D) organotypic cultures. Organoids of normal intestinal epithelium were isolated as we have previously described 24, 25 and cultured in Matrigel matrix (Corning Inc, Corning, NY, USA; Product No #354230) by a modification of a previously described method 26. Caco‐2 cells were also grown as monolayers, as were other cell types.

### Stable and transient transfections

We carried out mammalian SiRNA and plasmid DNA transfections using RNAiMAX and X‐tremeGENE transfection reagents (Thermo Fisher, Dublin, Ireland), respectively, as we have described previously 27. Lentiviral vector transfections were conducted using Lipofectamine 2000 (Thermo Fisher) according to the manufacturer's protocols. Stable clones were selected in blasticidin (Thermo Fisher). Overexpression of PLK4 encoded by the lentiviral system was induced by doxycycline treatment 28.

### Inhibition of intracellular protein–protein interactions

To study the biological effects of ezrin–NHERF1 interactions, cells were incubated with a cell‐permeant disruptor peptide of the ezrin binding domain in NHERF1 (KERAHQKRSSKRAPQMDWSKKNELFSNL) 29 or a control non‐targeting peptide (KERAHQKRSSKRAPQMDASKANELASNL). The peptides were synthesized by EZBiolab, Carmel, IN, USA.

### Fluorescent in situ hybridization (FISH) assays of chromosome segregation

In separate experiments, two‐colour FISH assays were performed in separate experiments using centromeric probes for chromosomes 1 (green) and 2 (red) or chromosome 19 (red) in separate experiments (Carl Zeiss, Cambridge, UK; XCP Human WCP probes). Assays of chromosome 19 mis‐segregation into micronuclei were also conducted 30.

### Confocal imaging

Assays of cell cortex dynamics, centrosome disposition, mitotic spindle orientation and geometry, nuclear pleomorphism, and multicellular patterns were conducted using a Leica SP5 confocal microscope, with an HCX PL APO lambda blue 63× 1.40 oil immersion objective at 1× or 2× zoom, as we have described previously 27.

### Human tumour samples

Anonymized formalin‐fixed, paraffin‐embedded (FFPE) colorectal primary tumours from previously described study cohorts 31 were released from the Northern Ireland Biobank (NIB), which has ethical approval to collect, store, and distribute anonymized tissue samples to researchers by an approved protocol.

Full details of cell culture methods, transfection, FISH assays, Ez/Nhe pbi and control peptide sequences, confocal imaging, human tumour samples, and ethical approval reference numbers are provided in the supplementary material, Supplementary materials and methods.

## Results

### Dynamics of ezrin cap formation

**Figure 1:**
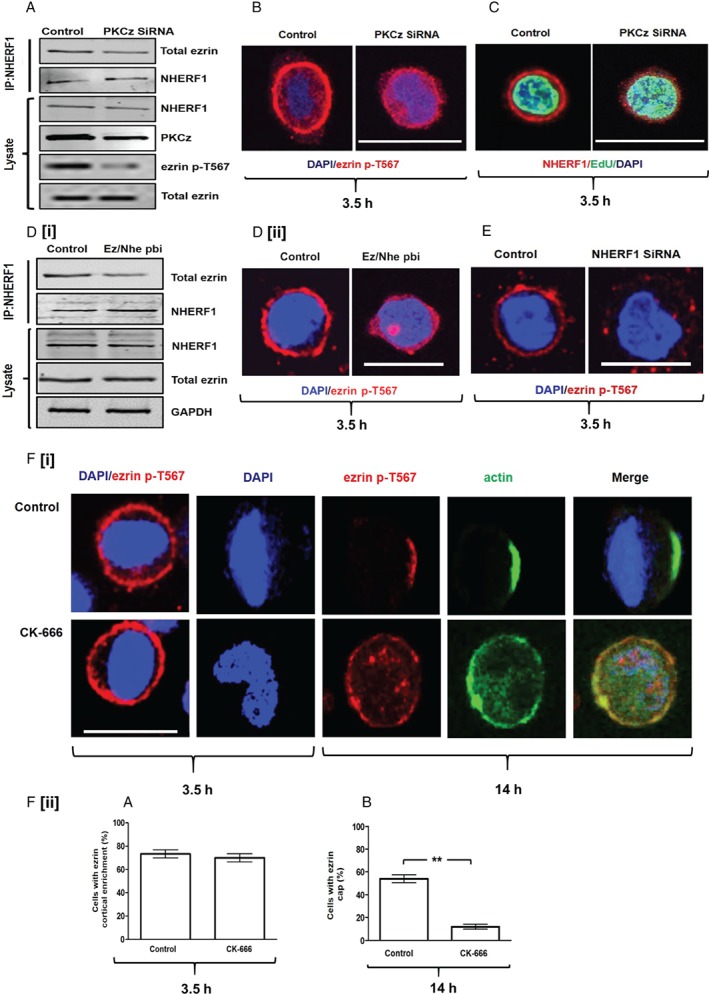
Dynamics of ezrin cap formation. (A) Co‐immunoprecipitation (CoIP) assays of total ezrin binding to NHERF1 in Caco‐2 cells transfected by control non‐targeting (NT) siRNA or PKCz siRNA. (B) Ezrin p‐T567 cortical recruitment in Caco‐2 cells transfected by control (NT) or PKCz siRNA. Assays at 3.5 h after plating. (C) NHERF1 cortical recruitment in Caco‐2 cells transfected by control (NT) or PKCz siRNA. For all cortical recruitment studies, cells were synchronized in G_0_/G_1_. At 3.5 h, most cells expressed the S‐phase marker EdU. Fifty EdU‐expressing cells were randomly selected and assessed in triplicate for each experimental condition. (D[i]) Co‐immunoprecipitation assays of total ezrin/NHERF1 interaction in Caco‐2 cells treated with a scrambled peptide (control) or the ezrin/NHERF1 peptide binding inhibitor (Ez/Nhe pbi). (D[ii]) Confocal assays of ezrin p‐T567 cortical enrichment in Caco‐2 cells treated with scrambled peptide (control) or Ez/Nhe pbi at 3.5 h after plating. (E) Confocal assays of ezrin p‐T567 cortical enrichment in Caco‐2 cells transfected by control non‐targeting (NT) siRNA versus NHERF1 siRNA KD at 3.5 h after plating. (F[i]) CK‐666 treatment (100 μm 36) effects on ezrin cortical recruitment at 3.5 h (column 1) and on ezrin and actin cortical cap formation at 14 h (columns 2–5). (F[ii]) (A) Summary effects of CK‐666 versus vehicle only control on ezrin p‐T567 cortical recruitment at 3.5 h (p = NS) and (B) on ezrin cap formation at 14 h (**p = 0.01). Analysis by paired Student's t‐test. Staining: DAPI (blue), ezrin p‐T567 (red), NHERF1 (red), EdU (green), actin (green). Scale bar = 20 μm.

Because PKCz regulates ezrin accumulation in the blastomere cell cortex 17 and ezrin/NHERF1 interaction controls cortical retention of ezrin 23, we investigated PKCz control of ezrin/NHERF1 interaction and ezrin cortical recruitment in Caco‐2 cells. To investigate PKCz regulation of ezrin/NHERF1 interaction, we immunoprecipitated NHERF1 from control and PKCz siRNA knockdown (KD) lysates. SiRNA PKCz KD led to reduced co‐immunoprecipitations of total ezrin by NHERF1 (Figure 1A and supplementary material, Figure S1A). PKCz siRNA KD or treatment by a PKCz pseudo‐substrate inhibitor (PKCzI) suppressed ezrin phosphorylation at T567 (supplementary material, Figure S1B–D), a key conformational switch 32 that enables ezrin/NHERF1 binding 33 and ezrin cortical enrichment 10. After cortical recruitment, ezrin becomes progressively restricted to form a pericentrosomal cap that anchors the interphase centrosome or clusters supernumerary centrosomes 10 (supplementary material, Figure S1E). To study ezrin cortical recruitment and temporal restriction of ezrin within the cortex to form the cap, we synchronized Caco‐2 cells in G_0_/G_1_ by serum starvation and conducted confocal microscopy assays. Here, we show ezrin cortical recruitment and cap formation at 3.5 and 14 h after plating, respectively (supplementary material, Figure S1F[i], [ii]). In addition, cortical dynamics of active ezrin p‐T567 parallels that of total ezrin (supplementary material, Figure S1F[i], [ii]). Since ezrin p‐T567 was more easily detectable, it was assessed in most confocal experiments. SiRNA PKCz KD inhibited cortical recruitment of ezrin p‐T567 (Figure 1B and supplementary material, Figure S1G) and NHERF1 (Figure 1C and supplementary material, Figure S1H). We used the S‐phase marker 5‐ethynyl‐2'‐deoxyuridine (EdU) to ensure equivalent cell cycle phases in experimental groups for NHERF1 studies (Figure 1C and supplementary material, Figure S1H). Merlin and ezrin are closely related 34 and PKCzI treatment suppressed merlin cortical recruitment (supplementary material, Figure S1I). PKCzI treatment reduced the percentage of Caco‐2 cells showing cortical localization of merlin from 32.7 ± 2.90% in control cells to 13.3 ± 2.0% after PKCzI treatment (*p* = 0.02). To investigate the role of ezrin/NHERF1 interaction in ezrin cortical recruitment, we used a specific ezrin/NHERF1 peptide binding inhibitor (Ez/Nhe pbi) 29 and conducted NHERF1 siRNA KD studies. Ez/Nhe pbi treatment inhibited the interaction between total ezrin and NHERF1 (Figure 1D[i] and supplementary material, Figure S1J) and suppressed ezrin p‐T567 cortical recruitment (Figure 1D[ii] and supplementary material, Figure S1K). Furthermore, NHERF1 siRNA KD (supplementary material, Figure S1L) also inhibited ezrin p‐T567 cortical recruitment (Figure 1E). Transfection of NHERF1 siRNA induced a fold reduction of NHERF1 protein expression of 0.55 ± 0.07 (*p* = 0.03) and reduced the percentage of cells with ezrin p‐T567 cortical recruitment from 73.3 ± 4.7% in control cells transfected with non‐targeting (NT) siRNA to 34.6 ± 4.1% in NHERF1 siRNA transfectants (*p* = 0.018).

Following ezrin cortical enrichment, mechanisms dependent on actin filaments, merlin, and α‐catenin drive ezrin cortical restriction to form the pericentrosomal cap 10. Because ezrin localization within the microvillus cortex can be driven by Arp2/3‐mediated actin treadmilling 35, we suppressed Arp2/3 using the specific inhibitor CK‐666 36. Here, we show that CK‐666 treatment did not affect ezrin cortical recruitment at 3.5 h but inhibited formation of both the ezrin cap and the actin cap at 14 h (Figure 1F[i], [ii]). Both merlin and NHERF1 decorated the entire circumference of the cell cortex at 14 h (supplementary material, Figure S1I, M). Because siRNA PKCz KD or disruption of ezrin/NHERF1 binding by peptide inhibitor treatment or siRNA NHERF1 KD (supplementary material, Figure S1L) suppressed ezrin cortical recruitment (Figure 1B, D[ii], E and supplementary material, Figure S1G, K), we tested the effects of these interventions on ezrin cortical cap formation. All of these interventions suppressed ezrin cap formation (data shown for PKCz siRNA and Ez/Nhe pbi treatment only; supplementary material, Figure S1N). The percentages of cells with ezrin cap formation were 66.70 ± 4.41% (control) versus 32.0 ± 2.0% (PKCz siRNA) versus 7.0 ± 1.53%; (Ez/Nhe pbi treatment) (*p* = 0.008 or *p* = 0.009, respectively). Collectively, these data support a two‐stage process of ezrin cap formation. Firstly, PKCz promotes ezrin phosphorylation to enhance ezrin/NHERF1 binding and ezrin cortical recruitment. Secondly, processes dependent on merlin 10 and Arp2/3 drive ezrin cortical restriction. Perturbation of either ezrin cortical recruitment or restriction impedes ezrin cap formation.

### Effects of ezrin/NHERF1 interaction on multicellular morphogenesis

**Figure 2:**
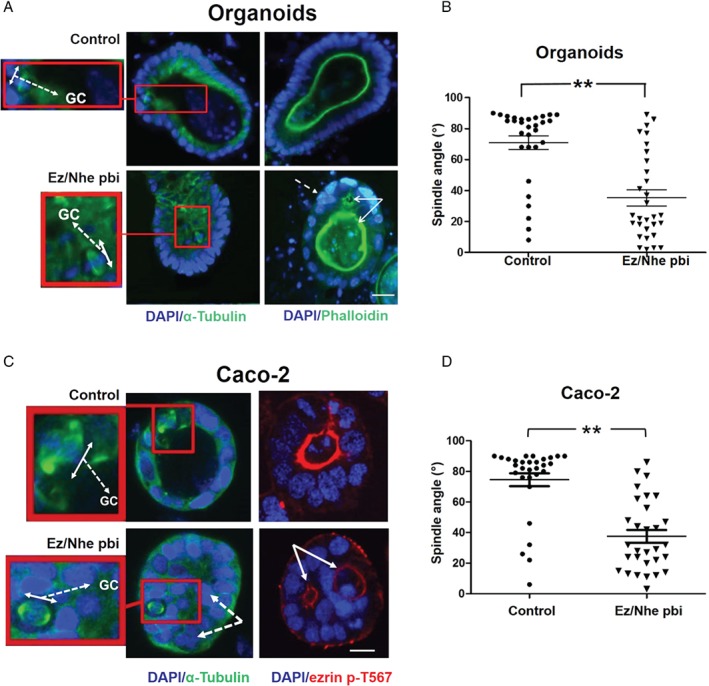
Effects of ezrin/NHERF1 interaction on multicellular morphogenesis. (A) Intestinal organoids. The left and right panels were stained to show mitotic spindle architecture and lumen formation, respectively. The left panels show bipolar spindle orientation in control and Ez/Nhe pbi‐treated organoids. High‐power spindle views (insets with red border) show the orientation angles (interrupted white arrows) of spindle planes (double‐headed white arrows) towards gland centres (GC). The right panels show lumen formation and epithelial configurations in control versus Ez/Nhe pbi‐treated organoids. Multiple lumens and early epithelial stratification are indicated by solid and interrupted white arrows, respectively, in Ez/Nhe pbi‐treated organoid cultures. (B) Summary spindle angles relative to GCs in control versus Ez/Nhe pbi‐treated organoids shown in A. **p < 0.01; paired Student's t‐test (n = 30 mitotic cells per experimental group). (C) Organotypic 3D Caco‐2 cultures. The left and right panels are stained to show mitotic spindle architecture and lumen formation, respectively. The left panels show bipolar spindle orientation in control and Ez/Nhe pbi‐treated Caco‐2 cultures. High‐power spindle views (insets with red border) show orientation angles (interrupted white arrows) of spindle planes (double‐headed white arrows) towards gland centres (GC). The right panels show lumen formation and epithelial configurations in control versus Ez/Nhe pbi‐treated Caco‐2 cultures. Multiple lumens and epithelial stratification are indicated by solid and interrupted white arrows, respectively, in Ez/Nhe pbi‐treated Caco‐2 cultures. (D) Summary spindle angles relative to GCs in control versus Ez/Nhe pbi‐treated Caco‐2 cultures shown in C. **p < 0.01. Analyses by paired Student's t‐test (n = 30 mitotic cells per experimental group). Staining: DAPI (blue), α‐tubulin (green), phalloidin (green), ezrin p‐T567 (red). Scale bar = 20 μm. Assays at 4 days of culture.

The ezrin cap controls mitotic spindle dynamics 10 that guides multicellular morphogenesis by well‐characterized biological mechanisms 7, 37. Here, we investigated ezrin/NHERF1 interactive effects on spindle dynamics and multicellular assembly in physiological and cancer models. We used organoids formed from primary intestinal cells 38 and 3D Caco‐2 organotypic CRC model systems 39. Acute perturbation of ezrin/NHERF1 interaction by peptide inhibitor treatment induced common effects of bipolar spindle misorientation, multi‐lumen formation, and epithelial stratification (Figure 2A–D and supplementary material, Figure S2A, D) that collectively induce cribriform multicellular morphology 31. To assess the effects on cellular phenotypes, we assessed nuclear roundness scores and nuclear size as indicators of pleomorphism 40. While suppression of ezrin/NHERF1 interaction reduced the nuclear roundness scores in both models (supplementary material, Figure S2B, E), it affected the nuclear size only in the Caco‐2 cells (supplementary material, Figure S2C, F). While ezrin and NHERF proteins have important roles in the organization of cell membrane domains and cell–cell and cell–extracellular matrix (ECM) communication 41, our studies reveal that ezrin/NHERF1 interaction is fundamental to morphogenic trajectories involving epithelial shape, configuration, spatial rearrangements, and luminogenesis in physiological and cancer states through control of bipolar mitotic spindle orientation.

### Effects of PKCz on mitotic spindle architecture in cells with extra centrosomes

**Figure 3:**
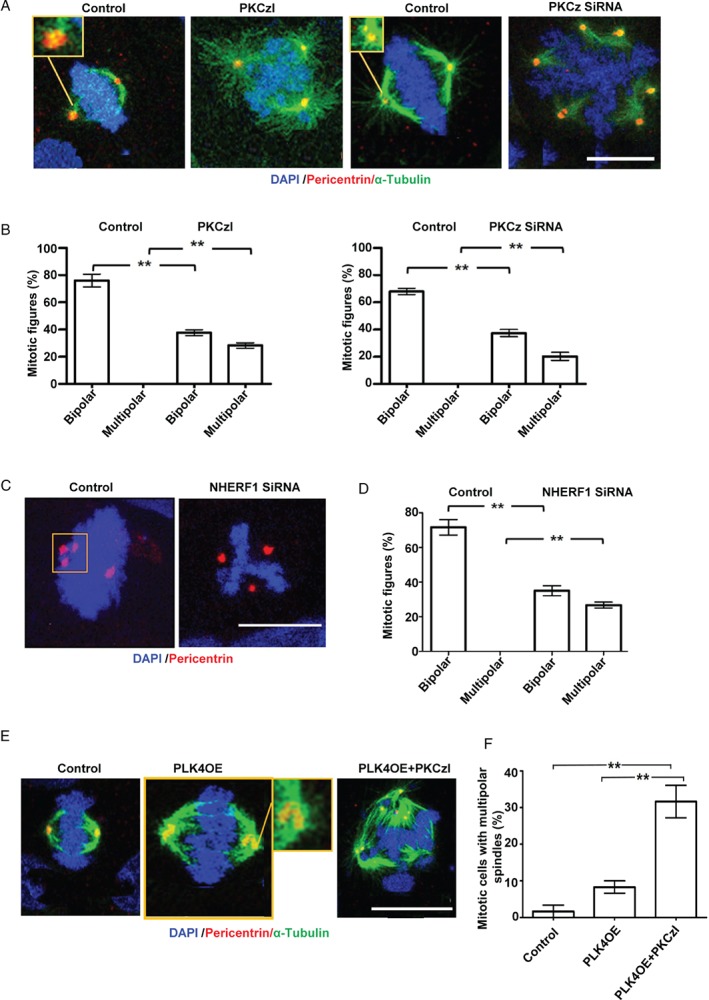
Effects of PKCz on mitotic spindle architecture in cells with extra centrosomes. (A) Centrosome clustering (insets with yellow borders) and spindle architecture in control, PKCzI‐treated (1 μm) or PKCz siRNA‐transfected Caco‐2 cells. (B) Summary mitotic spindle architecture data in control versus PKCzI‐treated Caco‐2 cells shown in A (bipolar: **p = 0.001; multipolar: **p = 0.002) and in control (NT siRNA) versus PKCz siRNA‐transfected Caco‐2 cells shown in A (**p = 0.002 for bipolar and multipolar). (C) Centrosome clustering (inset) in control versus NHERF1 siRNA‐transfected Caco‐2 cells. (D) Summary spindle architecture data in control versus NHERF1 siRNA‐transfected Caco‐2 cells shown in C (bipolar: **p = 0.002; multipolar: **p = 0.001). (E) Centrosome clustering (insets) and spindle architecture in control, PLK4OE, and PLK4OE + PKCzI‐treated Caco‐2 cells. (F) Summary multipolar spindle architecture data in control, Caco‐2 versus PLK4OE versus PLK4OE + PKCzI‐treated cells shown in E; Caco‐2 versus PLK4OE versus PLK4OE + PKCzI‐treated cells shown in E, **p < 0.001; control versus PLK4OE = NS, ANOVA, Tukey's post hoc test (n = 100 mitotic cells in triplicate, expressed as %). Monopolar or indeterminate mitotic figures were counted but not analysed. Staining: DAPI (blue), pericentrin (red), α‐tubulin (green). Scale bars = 20 μm.

Increased centrosome number is a common cancer characteristic 16, 42. Variable percentages of cells in Caco‐2 and other cancer lines contain extra centrosomes 14. In Caco‐2 cells, clustering of extra centrosomes at the ezrin cap enables bipolar spindle assembly 10. To investigate the role of PKCz in these processes, we conducted functional inhibition and/or siRNA knockdown (KD) studies against endpoints of multipolar spindle frequency and/or centrosome clustering. We investigated Caco‐2, U2OS, and B549 cancer cells, which are known to cluster extra centrosomes 10. PKCzI treatment or PKCz siRNA KD promoted the development of multipolar mitotic spindles in Caco‐2 cells (Figure 3A, B). These interventions also suppressed centrosome clustering, not only in Caco‐2 cells but also in U2OS cells (supplementary material, Figure S3A–C). To investigate the role of ezrin/NHERF1 interactions downstream of PKCz in these processes, we conducted siRNA KD studies or inhibited protein–protein interactions. SiRNA NHERF1 KD inhibited centrosome clustering (Figure 3C and supplementary material, Figure S3D) and promoted multipolar spindle architecture in Caco‐2 cells (Figure 3C, D). In keeping with this finding, siRNA NHERF1 KD also suppressed centrosome clustering in B549 cells (supplementary material, Figure S3E, F). Direct suppression of ezrin/NHERF1 interaction by peptide inhibitor treatment also prevented clustering and induced multipolar spindle formation in Caco‐2 cells (supplementary material, Figure S3G, H).

Although extra centrosomes are causally implicated in multipolar spindle formation, the relationship appears non‐linear 16. To investigate the association between extra centrosomes, PKCz, and spindle defects, we forced centrosome amplification in Caco‐2 and in chromosomally stable, near‐diploid HCT116 cells by stable overexpression of PLK4 15 (supplementary material, Figure S3I, J). PLK4 overexpression (PLK4OE) increased the percentages of Caco‐2 and HCT116 cells with extra centrosomes (supplementary material, Figure S3K, L). While PLK4OE caused only a modest increase in the frequency of multipolar spindle formation in Caco‐2 cells, PLK4OE combined with PKCz functional inhibition induced a substantively higher frequency of the multipolar spindle phenotype (Figure 3E, F). Taken together, these data show that PKCz ameliorates the effects of centrosome amplification via ezrin/NHERF1 interactions, interphase centrosome clustering at the ezrin cap, and suppression of multipolar spindle formation in cancer cells.

### Effects of PKCz on chromosome segregation in cells with extra centrosomes

**Figure 4:**
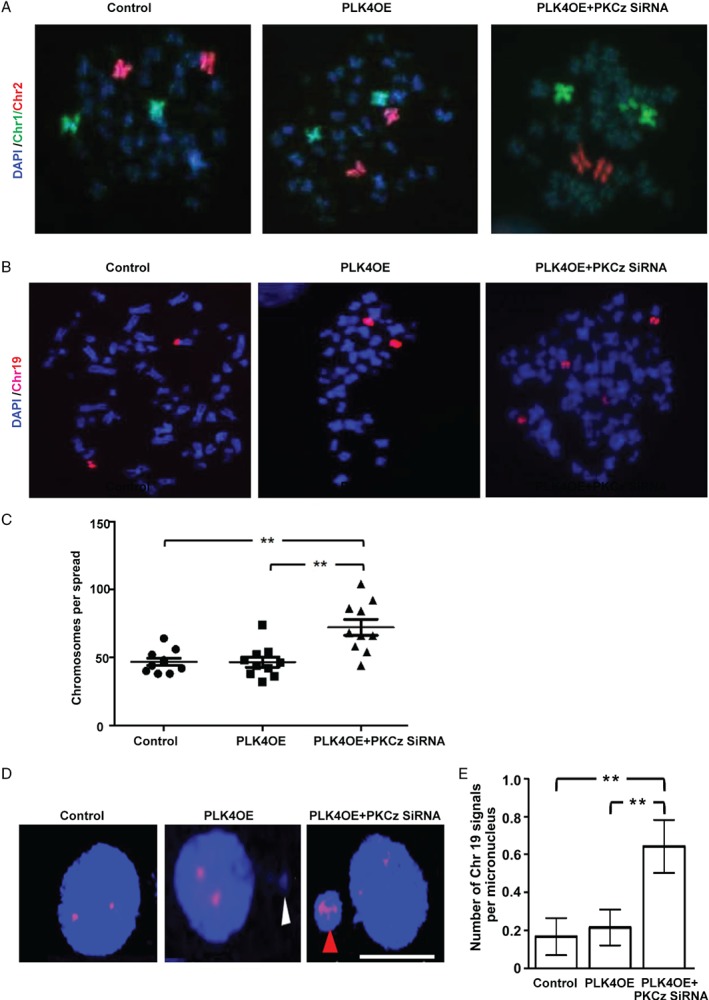
Effects of PKCz on chromosome segregation in cells with extra centrosomes. (A) Chromosome (Chr) 1 (green) and 2 (red) signals in control Caco‐2 transfected with empty vector only versus PLK4OE versus PLK4OE + PKCz siRNA‐transfected Caco‐2 cells. Chromosome fluorophores were counterstained against the DAPI DNA stain (blue). Note 2 × Chr1 and 2 × Chr2 signals in control and PLK4OE cells but 3 × Chr1 signals in Caco‐2 PLK4OE + PKCz siRNA‐transfected cells. We analysed 20 spreads per experimental condition. We found >2 Chr1 and/or >2 Chr2 signals in 1/20 control Caco‐2 and Caco‐2 PLK4OE spreads each. However, 4/20 Caco‐2 PLK4OE‐PKCz siRNA spreads showed >2 Chr1 and/or >2 Chr2 signals on FISH assay. (B) Chromosome 19 (red) signals in control Caco‐2 versus PLK4OE versus PLK4OE + PKCz siRNA‐transfected Caco‐2 cells (n = 30 cells per spread). We found >2 Chr19 signals in 9/30 control Caco‐2, 10/30 PLK4OE, and 14/30 Caco‐2 PLK4OE + PKCz siRNA‐transfected Caco‐2 cells. (C) Total chromosome number per spread in control Caco‐2 versus PLK4OE versus PLK4OE + PKCz siRNA‐transfected Caco‐2 cells, **p < 0.01; ANOVA; Tukey's post hoc test; control Caco‐2 versus PLK4OE = NS (n = 10 spreads per experimental condition). (D) Chromosome 19 (red) signals and micronuclei in control Caco‐2 versus PLK4OE versus PLK4OE + PKCz siRNA‐transfected Caco‐2 cells. Micronuclei lacking or containing a Chr19 signal are indicated by white or red arrowheads, respectively. (E) Summary of Chr19 signals per micronucleus in control Caco‐2 versus PLK4OE versus PLK4OE + PKCz siRNA‐transfected Caco‐2 cells, **p = 0.011; ANOVA with Tukey's post hoc test (Caco‐2 control versus Caco‐2 PLK4OE = NS). Scale bar = 20 μm.

The properly assembled bipolar mitotic spindle coordinates intracellular forces that drive equal genome partitioning 16. Conversely, multipolar spindle formation invokes segregation error either by progression through anaphase or by activation of different centrosome clustering mechanisms during metaphase that associate with merotelic attachments, mis‐segregation, and chromosomal instability (CIN) 43. To investigate the role of PKCz in chromosome segregation in cells with extra centrosomes, we conducted PKCz siRNA KD studies in stable PLK4‐overexpressing Caco‐2 (Caco‐2 PLK4OE) cells. We conducted FISH assays of chromosomes 1 and 2 because these chromosomes are large and suitable for assessment of chromosomal rearrangements. Conversely, we also studied chromosome 19 because it is small, frequently found in micronuclei 30, and contains CRC susceptibility loci 44. Here, we show that siRNA KD of PKCz in Caco‐2 PLK4OE cells (supplementary material; Figure S4A, B) induced aneuploidy of chromosome 1 (Figure 4A), chromosome 19 (Figure 4B), and increased total chromosome number (Figure 4C). We found micronuclei in 24/300 control versus 28/300 PLK4OE versus 42/300 PLK4OE + PKC siRNA‐transfected Caco‐2 cells. Some micronuclei contained chromosome 19 signals (Figure 4D). SiRNA PKCz KD increased chromosome 19 signals within micronuclei in Caco‐2 PLK4OE cells (Figure 4E). Taken together, these data indicate that PKCz knockdown in cells with extra centrosomes induces errors in genome partitioning including CIN and chromosome mis‐segregation into micronuclei.

### Relationships between mitotic spindle geometry and multicellular morphology in 3D organotypic CRC cultures

**Figure 5:**
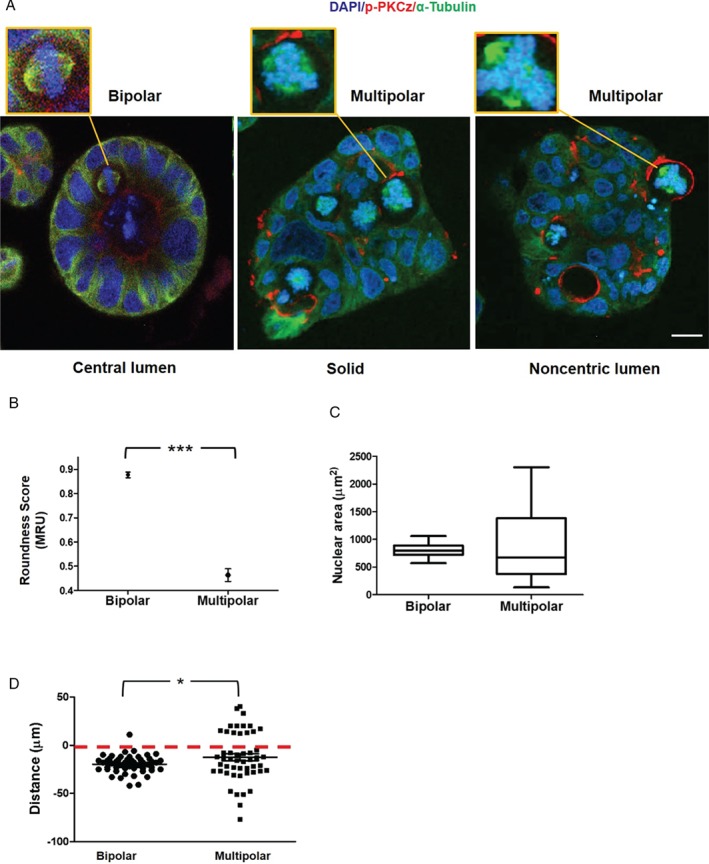
Relationships between mitotic spindle geometry and multicellular morphology in 3D organotypic CRC cultures. (A) Confocal assays of spindle architecture (insets) and multicellular morphology in 3D organotypic cultures. Control Caco‐2 cultures with appropriately orientated bipolar spindles (left panel) had regular 3D morphology with single central lumens surrounded by a uniform apical membrane and columnar epithelial monolayers. SiRNA knockdown of PKCz in 3D Caco‐2 PLK4OE cultures induced multipolar spindle formation and solid cell‐filled 3D structures with dispersed apical membrane foci. These cultures either lacked any lumen (middle panel) or had aberrant noncentric lumens lying outwith gland centres, surrounded by atypical epithelium (right panel). Cells with multipolar spindles extended across the basal interface with extracellular matrix (ECM) more frequently than cells with bipolar spindles (see D). (B) Nuclear ‘roundness’ scores in glands with bipolar versus multipolar spindles. ***p < 0.001; paired Student's t‐test. A score of 1 MRU denotes a perfect circle 70 (n = 60 cells from glands containing bipolar or multipolar spindles). (C) Range of nuclear size in glands with bipolar versus multipolar spindles (p < 0.01; Levene's test; n = 30 bipolar or multipolar cells). (D) Summary extension of cells with bipolar or multipolar spindles across the ECM interface (denoted by red interrupted line). Distances between spindle midpoints and the ECM interface were assessed. Positive or negative values were assigned for direction of extensions into or away from the ECM, respectively. Positive distance values (into the ECM) in multipolar versus bipolar spindles = 14/50 versus 1/50; *p = 0.03; paired Student's t‐test. Staining: DAPI blue, for nuclear DNA; p‐PKC red, for apical membranes, α‐tubulin green, for microtubules. Assays at 4 days of 3D culture.

In 3D cancer models, morphological adaptations are partly driven by bipolar mitotic spindle dynamics through control of abscission, cytokinesis 45, and multicellular assembly 7, 21. Conversely, associations between multipolar spindle architecture and cancer morphology remain unclear. In this study, interventions that induced the formation of multipolar spindles in Caco‐2 PLK4OE cell monolayers also did so in 3D Caco‐2 cultures. For example, siRNA PKCz KD or Ez/Nhe pbi treatment induced multipolar spindle formation in 3D Caco‐2 PLK4OE glandular structures (glands) (Figure 5A and supplementary material, Figure S5A, B). Multipolar spindle formation induced by siRNA PKCz KD was associated with the development of heterogeneous multicellular morphology. Phenotypic alterations included solid 3D multicellular structures with absent lumens or glands with multiple or noncentric lumens as well as atypical epithelial organization (Figure 5A). These changes were accompanied by nuclear pleomorphism evidenced by reduced nuclear roundness scores (Figure 5B) and a wide range of nuclear sizes (Figure 5C) in 3D glands. Furthermore, cells with multipolar spindles frequently extended across the basal interface with ECM in 3D cultures (Figure 5A, D). While Ez/Nhe pbi treatment induced multipolar spindle formation, it also suppressed growth of 3D Caco‐2 PLK4OE glands and thus hampered analysis of multicellular morphology. Collectively, our studies show that multipolar spindle formation induced by PKCz knockdown in cells with extra centrosomes induced CIN, nuclear pleomorphism, aberrant multicellular morphology, and spatial outgrowth of genomically unstable cells across the ECM interface. In combination, these phenotypes are evocative of aggressive, high‐grade CRC.

### Translational studies in archival colorectal cancer

**Figure 6:**
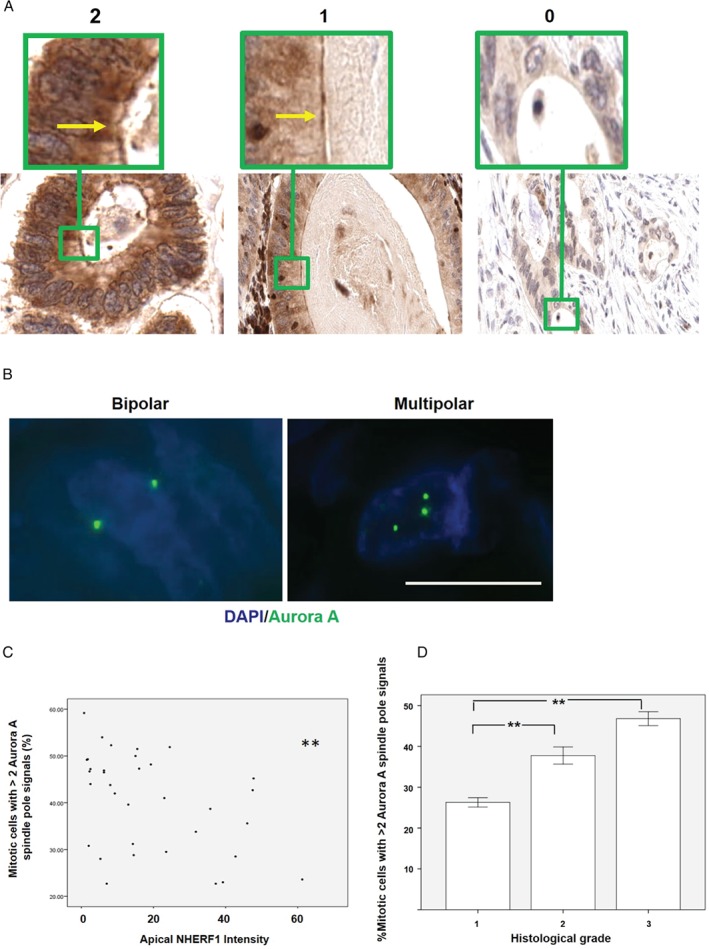
Translational studies in archival colorectal cancer. (A) IHC assay of NHERF1 apical expression in archival CRC. Sections at 50× objective magnification with scores of 2, 1, and 0, respectively. Apical localization of NHERF1 is indicated by yellow arrows in high‐power insets (green borders). (B) Cells with 2 or >2 Aurora A spindle pole signals in archival CRC sections indicative of bipolar or multipolar spindles, respectively 46. Objective magnification ×63. Staining: DAPI (blue), Aurora A (green). (C) Relationship between multipolar spindle formation (mitotic cells with >2 Aurora A spindle pole signals) and apical NHERF1 intensity (r = − 0.452); **p = 0.007; Pearson's test; Aurora A spindle pole signals assessed by IF in 180 ± 72 mitotic cells per tumour section in 35 CRCs. (D) Relationship between multipolar spindle frequency and cancer grade, **p = 0.005. ANOVA; Tukey's post hoc test. Multipolar spindles assessed as % of all mitotic figures. Scale bar = 20 μm.

Our findings link perturbations of interphase centrosome anchoring at the cell cortex to cancer phenotype anomalies in human CRC model systems. To integrate analyses from CRC models with primary human tumours, we conducted immunohistochemical (IHC) and immunofluorescence (IF) studies in archival CRC tissues. Apical NHERF1 intensity provides a robust readout of PKCz morphogenic activity in 3D cultures and has been used previously as an indirect readout in archival CRCs 39. Here, we investigated NHERF1 intensity in two CRC sample collections. Sample A comprised 35 whole tumour sections and five matched normal mucosa specimens, and sample B was a tissue microarray (TMA) comprising 309 tumour CRC specimen cores, derived from 92 CRCs 31. In sample A, we assayed apical NHERF1 intensity by IHC (Figure 6A) and mitotic spindle architecture by Aurora A IF assays of spindle poles (Figure 6B) 46. Apical NHERF1 intensity was inversely related to the frequency of mitotic cells with multipolar spindle architecture (Figure 6C), defined by more than two Aurora A‐positive spindle pole signals (Figure 6B), in CRC sections. In view of the small size of sample A, we also conducted semi‐quantitative assays of apical and total NHERF1 intensity by IHC in TMAs of sample B. We found a positive correlation between total and apical NHERF1 intensity (*r* = 0.504; *p* < 0.01), although apical NHERF1 scores had a stronger inverse relationship with lymph node metastases (*p* < 0.001; data not shown). We found that multipolar spindle frequency defined by Aurora A IF in CRC tissue sections was directly related to tumour grade (Figure 6D). Hence, these studies show that defective apical localization of NHERF1, a key component of centrosome anchoring machinery, is associated with multipolar spindle architecture and high‐grade morphology in human CRC.

In summary, we have shown that impaired cortical control of single or supernumerary interphase centrosomes provides unifying rationale for cell shape, genome segregation, and multicellular pattern phenotypes that characterize low‐ or high‐grade colorectal cancer (a graphic summary is shown in the supplementary material, Figure S6).

## Discussion

Mechanisms that integrate morphological and genomic phenotypes in colorectal cancer (CRC) represent a fundamental knowledge gap in pathology. In this study, we show that PKCz couples genome segregation to multicellular assembly by control of interphase centrosome anchoring. Furthermore, we reveal genomic, cytological, and morphological consequences of perturbation (summarized in the supplementary material, Figure S6).

Within the cell cortex, a polarized ezrin cap promotes anchoring and/or clustering of interphase centrosomes and guides mitotic spindle orientation and multicellular assembly 10. Here, we identify discrete steps in ezrin cap formation. Ezrin phosphorylation at T567 unmasks binding domains 32 and enhances ezrin/NHERF1 interaction 33 and ezrin cortical enrichment 10. We show that PKCz promotes ezrin T567 phosphorylation and increases ezrin/NHERF1 interaction. While ezrin/NHERF1 binding is required for maintenance of active ezrin at the cell cortex 23, the role of this molecular interaction in ezrin cortical recruitment remained unclear. This study shows that suppression of ezrin/NHERF1 interaction by peptide inhibitor treatment (Ez/Nhe pbi) blocked ezrin cortical recruitment. Furthermore, these effects on ezrin cortical recruitment were phenocopied by siRNA knockdown of NHERF1. Merlin (NF2) shares common ancestry with ezrin 47 and interacts with both ezrin 47 and NHERF1 48. We further show that functional inhibition of PKCz suppresses merlin cortical recruitment. Collectively, these findings indicate that PKCz promotes ezrin phosphorylation; enhances ezrin/NHERF1 binding; and promotes cortical enrichment of ezrin, NHERF1, and merlin.

In this study, we show cortical recruitment and cap formation of ezrin p‐T567 and total ezrin at 3.5 and 14 h, respectively, after plating. Within the cortex, ezrin restriction to form the cap depends on actin and merlin but is independent of myosin II motor activity 10. Unlike merlin 47, ezrin directly binds actin via a conserved C‐terminal binding domain 49, and ezrin and actin cap formation develop in parallel 10. We show that inhibition of the actin nucleator Arp2/3 by CK‐666 treatment 50 suppressed the formation of ezrin and actin caps without affecting ezrin cortical recruitment. Our findings are in accord with previous studies in the mouse oocyte, where Arp2/3 inhibition disrupted actin flow, suppressed cortical localization of actin at the cap, and perturbed spindle positioning 36. While Arp2/3‐mediated actin assembly can be suppressed by α‐catenin 51 and interaction of α‐catenin with merlin enables ezrin cap formation 10, mechanistic understanding is incomplete. Merlin is activated by plasma membrane phospholipids 52. To speculate, merlin activated by polarized plasma membrane phosphoinositides 52 could enhance ezrin cap formation via competitive binding to α‐catenin and release of Arp2/3‐driven actin flow from α‐catenin‐mediated inhibition.

In addition to genome partitioning, spindle dynamics also provide spatial directives for morphogenic processes 7, 53. To aid understanding of their role in cancer, we investigated morphological trajectories in physiological and cancer models and focused on commonalities as well as differences. Suppression of ezrin/NHERF1 interaction by inhibitory peptide treatment induced bipolar spindle misorientation, aberrant epithelial stratification, and multi‐lumen formation in both organoid and Caco‐2 CRC models. These phenomena led to cribriform morphology (CM) over longer‐term culture intervals of up to 12 days 31. While CM is regarded as a marker of malignant transformation in human colon 54, this study shows early features of this morphology in normal intestinal organoids when the ezrin/NHERF1 interaction was disrupted. Hence, these data suggest that cribriform morphogenesis is a consequence of bipolar spindle misorientation but is not necessarily restricted to malignant cells. In cancer, CM may reflect cumulative mutational silencing of core intrinsic regulators of mitotic spindle orientation. In the cancer model, acute perturbation of the ezrin/NHERF1 interaction had greater effects on nuclear roundness scores and nuclear area, possibly in association with intrinsic unidentified mutations.

In cells with extra centrosomes, the ezrin cap promotes clustering during interphase to enable bipolar spindle assembly 10. Conversely, failure of interphase centrosome clustering allows centrosome dispersal and multipolar spindle formation 16. To investigate the role of PKCz in centrosome clustering and spindle architecture, we forced centrosome amplification by PLK4 overexpression (PLK4OE) 15. Caco‐2 cells accommodated a large rise in PLK4‐induced centrosome number, with only a small increase in multipolar spindle formation. Conversely, functional inhibition of PKCz led to increased multipolar spindle frequency. To further investigate the connectivity between PKCz, ezrin, and NHERF1, we conducted siRNA knockdown studies or inhibited ezrin/NHERF1 interaction by peptide treatment. NHERF1 knockdown or inhibitory peptide treatment suppressed ezrin cap formation, prevented centrosome clustering, and promoted the multipolar spindle phenotype. Hence, PKCz controls mitotic spindle architecture by regulation of the ezrin/NHERF1 linkage and ezrin cortical dynamics.

Genome transmission and mitotic spindle assembly are intrinsically linked 16, 42. This study shows that impaired cortical anchoring of the normal interphase centrosome promotes bipolar spindle misorientation, abnormal epithelial configuration, and mislocalization of apical membrane markers. While these changes promote the development of cribriform morphology 31, they also enabled error‐free chromosome segregation in our model system. Our findings generally accord with previous studies in *Drosophila asterless* (*asl*) mutants 55, where impaired cortical anchoring of the interphase spindle pole induced abnormal spindle positioning, aberrant cell configuration, and mislocalization of cortical proteins 55. Furthermore, impaired anchoring of a single spindle pole during interphase induced only a slight compromise to the fidelity of chromosome segregation in the *Drosophila* model 55. Conversely, transition to multipolar spindle formation in the Caco‐2 system promoted chromosomal instability (CIN). Whole mis‐segregated chromosomes may become incorporated within micronuclei in inverse proportion to their size 30 and may drive chromothripsis 56, a major mutagenic phenomenon 57. We show that PLK4OE combined with PKCz knockdown increased whole chromosome number as well as the number of chromosome 19 signals within micronuclei. These data indicate that effective clustering of extra centrosomes during interphase by PKCz‐dependent cortical machinery inhibits multipolar spindle formation and suppresses CIN and chromosomal misincorporation into micronuclei that can trigger complex genomic rearrangements 56.

In cancer, aberrant morphology is classified within grading systems to provide the best‐established predictors of clinical outcome 58, 59. Key features of high‐grade aggressive cancer include gross nuclear pleomorphism, aberrant mitotic figures, and loss of glandular architecture 60, 61, 62. Here, we show that suppression of PKCz‐dependent cortical machinery in cells with extra centrosomes drove the development of these high‐grade cancer phenotypes in 3D cell model systems. All Caco‐2 PLK4OE glands with multipolar spindles induced by siRNA PKCz KD developed very abnormal morphology. None had a single central lumen and most comprised solid, cell‐filled structures with widely dispersed apical membrane foci. These foci varied in size, probably from differences in transapical secretion 8. In larger foci, the ectopic apical membrane enclosed discernible lumens that we termed noncentric, as they were not situated in gland centres. We described glands as cell‐filled if they contained only cells and no lumen‐like structures. Ez/Nhe pbi treatment drove multipolar spindle formation but also appeared to suppress the growth of Caco‐2 PLK4OE glands and thus hampered interpretation of multicellular morphology. Precise mechanisms of Ez/Nhe pbi growth suppression remain unclear but could be related to robust inhibition of ezrin cortical recruitment. Malignant cell detachment from the main tumour mass is a key metastatic process 58 and is a common histological feature in high‐grade cancer sections 63, 64. In a *Drosophila* tumour model, cell extrusion across basement membrane and early invasion can be driven by chromosomal instability (CIN) 65. In accord with those findings, we show that multipolar spindle formation promotes CIN and cell extension across the extracellular matrix interface in organotypic 3D CRC cultures.

Correlative analyses in archival human tumour samples may shed light on experimental discoveries. Apical NHERF1 intensity provides a readout of PKCz morphogenic signalling in 3D organotypic Caco‐2 glands and has previously been used as an indirect readout in paraffin‐fixed tissues 21. In the present study, apical NHERF1 IHC intensity was inversely related to multipolar spindle formation, defined by Aurora A IF 46. Furthermore, multipolar spindle frequency was directly associated with aberrant multicellular morphology of high‐grade CRC. Hence, defective PKCz cortical signalling reflected by low apical NHERF1 intensity 21 may underlie mitotic errors and aberrant multicellular morphology that characterize aggressive CRC.

This study has uncovered core molecular machinery that controls centrosome anchoring, mitotic spindle geometry, genome segregation, and multicellular assembly. Perturbation of these processes by diverse oncogenic pressures 10, 66, 67, 68, 69 may provide a phylogenetic basis for branched evolution of genomic and morphological phenotypes underlying cancer trajectories to more aggressive subtypes.

## Author contributions statement

RKD conducted signalling, centrosome, spindle, and multicellular morphogenesis assays; data analysis; and figure preparation. AJ conducted signalling, transfections, and some morphogenesis assays. JMcC and MBL conducted morphological assays and data analysis in archival cancer. JV and KS conducted or supervised metaphase squash chromosome stability and micronucleus assays. EE conducted infections with PLK4 lentiviral vectors and contributed extensively to writing. FCC conceived the study, designed experiments, analysed data, and wrote the manuscript with additional input from all co‐authors.

**SUPPLEMENTARY MATERIAL ONLINE**

**Supplementary materials and methods**

**Supplementary figure legends**

**Figure S1.** Dynamics of ezrin cap formation (supplementary)

**Figure S2.** Summary effects of ezrin/NHERF1 interaction on multicellular morphogenesis

**Figure S3.** Summary effects of PKCz on mitotic spindle architecture in cells with extra centrosomes

**Figure S4.** Effectiveness of siRNA PKCz knockdown

**Figure S5.** Spindle architecture in control versus Ez/Nhe pbi‐treated Caco‐2 cultures and associated quantitative data

**Figure S6.** Graphic summary – effects of defective centrosome anchoring on evolution of CRC morphological and/or genomic phenotypes

**Table S1.** Antibodies, suppliers, catalogue numbers, and dilutions used

## Supporting information


**Supplementary materials and methods**

**Supplementary figure legends**

**Figure S1.** Dynamics of ezrin cap formation (supplementary). **(A)** Quantification of total ezrin/NHERF1 binding shown in Figure 1A after PKCz siRNA KD normalized to control non‐targeting (NT) siRNA transfections = 0.66 ± 0.038; *p = 0.0123. (**B)** Immunoblots in Caco‐2 cells after PKCz pseudo‐substrate inhibitor (PKCzI 1 μm) 21 treatment or PKCz siRNA transfection versus vehicle only or non‐targeting siRNA controls. Quantification of ezrin p‐T567 ADU in western blots shown in **B** after PKCzI treatment **(C)** = 0.45 ± 0.05;**p = 0.009 or PKCz siRNA KD **(D)** = 0.54 ± 0.04; **p = 0.009. Values are normalized to control. (**E)** Schematic of ezrin cap formation in relation to interphase centrosome anchoring, replication, and clustering. Ezrin (red) is recruited from the cytosol to the cortex, where it becomes progressively restricted to form the ezrin cap, close to the interphase centrosome. The ezrin cap binds centrosomal astral microtubules (green) 10. Thus anchored to the cell cortex, the centrosome (orange) then replicates to generate one mother and one daughter centrosome (single curved arrow). In cancer, oncogenic processes drive abnormal centrosome replication (double curved arrow) to generate extra centrosomes (encircled) 14, 15. Extra interphase centrosomes are thus clustered at the ezrin cap 10. (**F)** Cortical recruitment and restriction of ezrin p‐T567 (**F[i])** and total ezrin (**F[ii])** in Caco‐2 cells. Intervals of 3.5 and 14 h after plating were suitable for assay of ezrin cortical recruitment and cap formation, respectively. (**G)** Effects of PKCz siRNA KD on ezrin p‐T567 cortical recruitment at 3.5 h shown in Figure 1B; *p = 0.012. (**H)** Effects of PKCz siRNA KD on NHERF1 cortical recruitment at 3.5 h shown in Figure 1C; **p = 0.001. Values represent % cells with ezrin or NHERF1 cortical recruitment normalized against control. (**I)** Merlin cortical localization in control or PKCzI treated Caco‐2 cells at 14 h after plating. (**J)** Effects of Ez/Nhe pbi versus scrambled peptide control on total ezrin/NHERF1 binding shown in Figure 1D[i]; *p = 0.012. (**K)** Effects of inhibitory peptide treatment on ezrin p‐T567 cortical recruitment at 3.5 h after plating shown in Figure 1D[ii]; **p = 0.0035. (**L)** Effects of NHERF1 siRNA KD versus NT siRNA on NHERF1 expression. (**M)** Confocal assays of ezrin and NHERF1 localization at 14 h after plating in Caco‐2 cells. NHERF1 does not localize at a cap. (**N)** Effects of PKCz siRNA KD (left panels) or Ez/Nhe pbi treatment (right panels) on ezrin cap formation in Caco‐2 cells at 14 h after plating. All analyses by paired Student's t‐test. Staining: DAPI (blue), ezrin p‐T567 (red), merlin (red), total ezrin (green). Scale bars = 20 μm.

**Figure S2.** Summary effects of ezrin/NHERF1 interaction on multicellular morphogenesis. **(A)** Summary of single lumen formation in control versus Ez/Nhe pbi‐treated organoids shown in Figure 2A;*p = 0.03 (n = 30 organoids per experimental condition in triplicate, expressed as %). (**B)** Nuclear ‘roundness’ scores in control versus Ez/Nhe pbi‐treated organoids [measured roundness units (MRU)]; *p = 0.02. (**C)** Nuclear area in control and Ez/Nhe pbi‐treated organoids shown in Figure 2A, p = NS. (**D)** Summary of single lumen formation in control versus Ez/Nhe pbi‐treated Caco‐2 glands shown in Figure 2C, **p = 0.004 (n = 30 Caco‐2 glands per experimental condition in triplicate, expressed as %). (**E)** Nuclear ‘roundness’ scores in control versus Ez/Nhe pbi‐treated Caco‐2 glands shown in Figure 2C, ***p < 0.001. (**F)** Nuclear area in control and Ez/Nhe pbi‐treated Caco‐2 cultures shown in Figure 2C, *p = 0.012; n = 100 cells per experimental condition in triplicate. All analyses by paired Student's t‐test.

**Figure S3.** Summary effects of PKCz on mitotic spindle architecture in cells with extra centrosomes. **(A)** Summary effects of PKCz siRNA KD versus control on centrosome clustering in Caco‐2 cells shown in Figure 3A (right panels), **p = 0.001. **(B)** Centrosome clustering (inset) in PKCzI‐treated U2OS cells versus control. (**C)** Summary effects of PKCzI versus control on centrosome clustering in U2OS cells shown in B, *p = 0.05. **(D)** Summary effects of NHERF siRNA KD on centrosome clustering in Caco‐2 cells shown in Figure 3C, *p = 0.03. (**E)** Centrosome clustering (inset) in NHERF1 siRNA‐transfected B549 cells versus control. (**F)** Summary effects of NHERF1 siRNA KD versus control on centrosome clustering in B549 cells shown in E;**p = 0.005. (**G)** Mitotic spindle architecture in Ez/Nhe pbi‐treated Caco‐2 cells versus control. (**H)** Summary effects of Ez/Nhe pbi treatment versus control on mitotic spindle architecture in Caco‐2 cells shown in G; bipolar: *p = 0.01; multipolar: **p < 0.001. Clustering was assessed in n = 100 cells with more than two centrosomes in triplicate. Spindle architecture was assessed in 100 mitotic cells in triplicate, expressed as a percentage. (**I)** Doxocycline‐inducible PLK4 overexpression (PLK4OE) in Caco‐2 and HCT116 cells. (**J)** Summary of doxycycline‐inducible PLK4 ADU in Caco‐2 and HCT116 cells shown in I. (**K)** Centrosome amplification of PLK4‐overexpressing (PLK4OE) Caco‐2 and HCT116 cells versus control. Assays at 24 h after doxycycline‐driven PLK4 overexpression. (**L)** Summary effects of PLK4OE on centrosome number in Caco‐2, **p = 0.006, and HCT116 cells **p = 0.007, shown in K. Analyses by paired Student's t‐test (A, C, D, F, L) or ANOVA (H). Staining: DAPI (blue), pericentrin (red), and α‐tubulin (green).

**Figure S4.** Effectiveness of siRNA PKCz knockdown. **(A)** PKCz expression after siRNA knockdown versus control in Caco‐2 PLK4OE cells. (**B)** Summary effects of PKCz siRNA KD versus control on PKCz ADU in Caco‐2 PLK4OE cells shown in A,** p = 0.008; paired Student's t‐test.

**Figure S5.** Spindle architecture in control versus Ez/Nhe pbi‐treated Caco‐2 cultures and associated quantitative data. **(A)** Spindle architecture (insets) in control versus Ez/Nhe pbi‐treated Caco‐2 cultures. (**B)** Summary effects of Ez/Nhe pbi treatment versus control on spindle architecture shown in A; bipolar: **p = 0.008; multipolar: **p = 0.003 (100 mitotic cells assessed in triplicate in each experimental condition). Analysis by ANOVA. Staining: DAPI (blue), pericentrin (red), α‐tubulin (green).

**Figure S6.** Graphic summary – effects of defective centrosome anchoring on evolution of CRC morphological and/or genomic phenotypes. **(i)** Ezrin cap (red) anchoring of the interphase centrosome through astral microtubule binding (green). Thus stabilized, the centrosome replicates to generate two normal or extra (encircled) centrosomes 10. **(ia1, ia2)** Bipolar spindle assembly and normal orientation with normal **(ia1)** or clustered **(ia2)** centrosomes. **(ib)** Correct bipolar spindle orientation, normal cleavage furrows, and appropriate apical membrane (AM; red) alignment. **(ic)** Lumen expansion driven by secretion (blue arrows). These steps enable appropriate multicellular assembly and gland formation 7. **(id, ie)** Representative images in the culture model and normal colon. **(ii)** Defective ezrin cap formation with a single interphase centrosome. Impaired anchoring of the interphase centrosome to the cell cortex drives bipolar spindle misorientation **(iia)** 55. In turn, bipolar spindle misorientation drives apical membrane AM (red) misalignment and aberrant planes of cell cleavage 7 **(iib).** Collectively, these processes induce cribriform multicellular morphology **(iic)** 31. (**iid, iie)** Representative images in the culture model and low‐grade colorectal cancer. **(iii)** Defective ezrin cap formation with extra centrosomes. Dispersal of multiple, unanchored centrosomes promotes transient multipolar spindle formation 16, 42. **(iiia)** Thus formed, most transient multipolar spindles are converted to misorientated, pseudo‐bipolar spindles by error‐prone metaphase clustering mechanisms, accompanied by chromosome lag (shown in the cartoon) 16 **(iiib1)**. A few cells with multipolar spindles undergo multipolar division to generate pleomorphic progeny 16 **(iiib2).** Segregation error associated with these processes promotes chromosomal instability (CIN). In the present study, we show whole chromosome (Chr) aneuploidy indicated by 3 × Chr1 (green) signals **(iiic1).** CIN arising from these mechanisms is accompanied by nuclear pleomorphism, gross multicellular perturbation, and extrusion of genomically unstable cells across the basal interface with the ECM, shown in the cartoon **(iiic2)** and culture model **(iiid)**. Insets show bipolar or multipolar spindle architecture. Extrusion of malignant cells from the main epithelial mass in high‐grade CRC is shown in **iiie.**

**Table S1.** Antibodies, suppliers, catalogue numbers, and dilutions used
