# Supplementary material for: Protein kinase C zeta suppresses low‐ or high‐grade colorectal cancer (CRC) phenotypes by interphase centrosome anchoring
Source: J Pathol. 2018 Mar 9;244(4):445–59. doi: 10.1002/path.5035 (PMC5873423; doi:10.1002/path.5035)
Supplement: Supplementary file 1 — Supplementary materials and methods [file PATH-244-445-s006.docx]

**Supplementary materials and methods**

Reference numbers refer to the main text reference list

**Reagents and antibodies**

Reagents were obtained from Sigma-Aldrich, UK, unless otherwise stated. Full details of the antibodies, suppliers, catalogue numbers, and dilutions used are shown in the supplementary material, Table S1. In brief, the antibodies used in this study were mouse anti-PKCz (Abcam, Cambridge, UK), rabbit anti-phospho-PKCz (T560) (Abcam), rabbit anti-PLK4 antibody (Abcam), mouse anti-NHERF1 (Lifespan Biosciences, Seattle, WA, USA), mouse anti-ezrin (Abcam), rabbit anti-phospho-ezrin (T567) (Abcam), rabbit anti-pericentrin (Abcam), mouse anti-α-tubulin (Abcam), FITC-phalloidin and FITC-α-tubulin (Sigma-Aldrich, Dorset, UK), mouse anti-neurofibromin (also known as merlin – Santa Cruz antibodies, Santa Cruz Biotechnology, Santa Cruz CA, USA), and mouse anti-GAPDH (glyceraldehyde-3-phosphate dehydrogenase; Abcam). These primary antibodies were used where appropriate in conjunction with LI-COR IR Dye680 (anti-rabbit) and IR Dye800 (anti-mouse) secondary antibodies for use with the LI-COR Infra-Red imaging systems (LI-COR Biosciences, Lincoln, NE, USA) in western blots or with Alexa Fluor 488 (anti-mouse) and Alexa Fluor 568 (anti-rabbit) (Molecular Probes, Thermo Fisher, Carlsbad, CA, USA) for fluorescence or confocal microscopy detection of mouse or rabbit primary antibody immunoglobulin (IgG), as appropriate. Anti-phospho-Aurora A antibodies were from BD Transduction Laboratories, Oxford, UK (Clone 4 IAK1, Cat No 610939). FITC-phalloidin and FITC-α-tubulin high affinity probes to F-actin and α-tubulin conjugated to the green fluorescent dye, fluorescein isothiocyanate (FITC), were obtained from Sigma-Aldrich. RNAiMAX and X-tremeGENE transfection reagents were purchased from Thermo Fisher (Dublin, Ireland) and Roche (Basel, Switzerland), respectively. Dharmacon Smart-Pool On-Target plus human PKCz siRNA (catalogue No L-003526-00-0005; Thermo Fisher Scientific) and non-targeting control siRNA oligonucleotides (catalogue No D-001810-01-05; Thermo Fisher Scientific) were used to suppress PKCz. Oligofectamine and Opti-Mem serum-free medium (Invitrogen) were mixed in a 1 : 3 ratio, incubated at room temperature for 5 min, and then added to siRNA, which was prediluted in Opti-Mem to a final concentration of 50 nm and then added to cultures. Cells were grown in 90 mm dishes and were transfected at 50–60% confluence by incubation in the above mixture. The medium was changed after 6 h and cells were harvested after 48 h. PKCz expression was assayed by western blot*.* We used a PKCz pseudo-substrate inhibitor (PKCzI) that has been extensively validated in various cell types [[71–73](#_ENREF_71)] (Sigma, p1614**,** MDL number [MFCD03458229](http://www.sigmaaldrich.com/catalog/search?term=MFCD03458229&interface=MDL%20No.&N=0&mode=partialmax&lang=en&region=GB&focus=product)) for inhibition of PKCzeta activity.

**Cell culture**

Caco-2, BT-549, and U2OS cells were obtained from ATCC, Middlesex, UK. HCT116 cells were a gift from Dr Todd Waldman (Georgetown University, USA). Caco-2 cells were cultured in DMEM supplemented with10% fetal calf serum (FCS), 1 mm non-essential amino acids, and 1 mm l-glutamine at 37°C in 5% CO_2_. HCT116 cells were cultured in McCoy’s 5A medium supplemented with10% FCS, 1 mm sodium pyruvate, and 1 mm l-glutamine. U2OS and BT-549 cells were cultured in DMEM with 10% FCS. In 3D cultures, Caco-2 cells and subclones stably transfected with empty vector (EV) only or doxycycline (Doxy)–inducible polo-like kinase 4 (PLK4) were mixed with Matrigel (40%), plated into a well of an eight-chamber slide, allowed to solidify for 30 min at 37°C, and subsequently overlaid with 400 μl of MEM (modified Eagle’s medium) supplemented with 10% FCS, 1 mm non- essential amino acids, and 1 mm l-glutamine per well, as we have previously described [[31](#_ENREF_31)].

**Co-immunoprecipitation (co-IP)**

Cells were lysed on ice in buffer containing 100 mm Tris–HCl (pH 7.5), 1% Triton X-100, 5 mm EDTA, 50 mm NaCl, 5 mm NaF, 1 mm Na_3_VO_4_, and protease inhibitor (Complete mini protease inhibitor cocktail, Sigma-Aldrich, product No 04693159001). Cell lysates were centrifuged (for 10 min at 15 000 × *g*) and 1000 µg of protein lysate was precleared overnight with control IgG and Protein A/G Sepharose beads (Santa Cruz Biotechnology, CA, USA). The protein was then immunoprecipitated with the appropriate antibody for 2 h at 4°C. The beads were collected by centrifugation, washed extensively, and finally resuspended in Laemmli sample buffer.

**Lentiviral vectors**

To generate the inducible PLK4 overexpression system, we used lentiviral vectors pLenti-CMV-TetR-Blast (#17492, Addgene) and pLenti-CMV/TO-Neo-Dest (#17292, Addgene), as previously described [[74](#_ENREF_74)]. These were provided as a gift by Dr S Godinho, Bart’s Cancer Institute, London. Stable clones were treated with a dose range of doxycycline for 48 h to optimize transgene expression for use in subsequent experiments.

**Imaging of cell monolayers and image analysis**

For assays of cortical protein expression and ezrin cap formation, cells were cultured in six-well plates. For transfections, cells were incubated in transfection reagents containing test or control constructs for 24 h. Cells were then harvested and placed in Matrigel-coated eight-well multichambers and imaged at intervals up to 14 h after plating. Cells were fixed with 2% paraformaldehyde (PFA) and then immunostained with appropriate antibodies at 4°C overnight. In our image analysis, cortical protein recruitment was defined as protein localization to the cell cortex beneath the cell surface, in excess of the protein quantity dispersed throughout the cytoplasm [[75](#_ENREF_75)]. Ezrin cap formation was defined as local ezrin accumulation to form a cap-like structure at one pole of the cell cortex of spherical cells, prior to the first cell division [[10](#_ENREF_10)]. Cortical protein expression, ezrin cap formation, centrosome number, disposition, and spindle architecture were imaged by confocal microscopy and quantified by ImageJ in triplicate for each experimental condition.

**Assessment of cortical architecture and ezrin cap**

We assessed ezrin p-T567 as a readout of active ezrin [[10](#_ENREF_10)]. Images were collected using fixed laser settings in the confocal microscope. Signal intensity within the cortex was quantified using ImageJ. Cortical localization assays of NHERF1 after non-targeting (NT) or PKCz SiRNA transfection were conducted using the cell cycle S-phase marker 5-ethynyl-2'-deoxyuridine (EdU) [[76](#_ENREF_76)], using the Click IT protocol (Thermo Fisher, Dublin, Ireland) following the manufacturer’s instructions.

**Assessment of centrosome clustering and spindle architecture**

In cultured cells, centrosomes and microtubules (MTs) were identified by confocal microscopy using anti-pericentrin and anti-α-tubulin antibodies, respectively, while chromosomal DNA was imaged using 4',6-diamidino-2-phenylindole (DAPI) staining. A bipolar mitotic spindle was defined by convergence of MTs towards each of two spindle poles, while a multipolar spindle was defined by more than two poles combined with abnormal DNA separation patterns. Centrosomes in excess of two per cell were defined as supernumerary. Mitotic cells were scored for clustered (more than one centrosome at spindle poles) or unclustered centrosomes and for multipolar spindles.

**Chromosome spreads and FISH analysis**

Control, transfected, and treated cells were incubated in 10 μg/ml Karyomax Colcemid solution (0.05 μg/ml; Thermo Fisher Scientific, Dublin, Ireland) for 18 h. Following this, cells were harvested and metaphases were collected by resuspending in hypotonic 75 mm KCl for 20 min at 37°C, followed by fixation for 20 min at 4°C in freshly prepared ethanol/glacial acetic acid, 3:1, v/v [[77](#_ENREF_77)]. After two more washes in this mixture, cells were dropped onto pre-warmed wet slides and air-dried at room temperature and aged at room temperature for 7 days. Aged slides were hybridized with whole chromosome fluorescence-labelled DNA probes (Carl Zeiss, Oxford, UK; XCP, Human, Whole-Chromosome Probes) directed to chromosome 1, chromosome 2, and chromosome 19 following the manufacturer’s protocol. DNA denaturation (72°C for 3 min) and hybridization (37°C for 8 h) were performed using the Vysis HYBrite chamber system (Abbott Diagnostics, Biosurplus, San Diego, CA, USA). Slides were washed with 0.4× SCC at 72°C for 2 min and then with 2× SCC and 0.05% Tween-20 at room temperature for 30 s, and mounted in Prolong Gold containing DAPI for chromosome counterstaining (Thermo Fisher, Dublin, Ireland). Slides were imaged using a Nikon Eclipse TiNS epifluorescence microscope, with a 63× objective.

**Intestinal organoid cultures**

C57B/6 wild-type mice (< 10 weeks old) were used for experiments. All animal procedures were conducted in accordance with local and national regulations. Organoids were isolated by modifications [[26](#_ENREF_26)] of previously described methods [[25](#_ENREF_25)]. In brief, murine intestines were opened longitudinally, cut into 5 mm fragments, and washed 7–10 times in 1× HBSS [low calcium, low magnesium (Gibco-BRL), 2% d-glucose, 0.035% NaHCO_3_] to remove all luminal contents. The fragments were then finely chopped with a scalpel and digested in HBSS solution containing collagenase and dispase I neutral proteases (Sigma-Aldrich, Dorset, UK) at 1 mg/ml for 20 min at room temperature on a shaking platform. Digestion was stopped by the addition of 30 ml of DMEM/F12 culture medium (Life Technologies, Renfrew, UK) supplemented with 5% FCS containing penicillin and streptomycin. Large fragments and muscle sheets were allowed to settle to the bottom of the flask. Supernatant containing the organoids was centrifuged for 3 min at 250 rpm, to pellet the organoids. The supernatant was removed and the organoid pellet was gently resuspended in 20 ml of the DMEM/F12 solution. The centrifugation step was repeated 5–6 times until the pellet contained a homogeneously sized organoid preparation. Organoids were resuspended in a 2× volume of Matrigel (growth factor-reduced, phenol red-free; BD Biosciences, Oxford, UK) supplemented with 50 ng/ml murine EGF, murine Noggin (100 ng/ml; Thermo Fisher, Dublin, Ireland), and 1 μg/ml human R-spondin (PeproTech, NJ, USA), as indicated for organoid culture [[26](#_ENREF_26)]. Eight-well multichambers were coated with a thin layer of undiluted Matrigel and allowed to solidify. Organoid preparations in Matrigel (100 μl suspension) were placed into each well and then overlaid with 250 μl/well culture medium (Dulbecco’s modified Eagle medium/F12) supplemented with penicillin/streptomycin, 10 mm HEPES, Glutamax supplements 1× N2, 1× B27 (ThermoFisher, Dublin, Ireland), 1 mm *N*-acetylcysteine (Sigma-Aldrich, Dorset, UK), 50 ng/ml murine EGF, Noggin (100 ng/ml), and 1 μg/ml human R-spondin [[26](#_ENREF_26)]. Organoids were cultured for 4 days.

**Confocal immunofluorescence microscopy of 3D cultures and organoids**

Embedded glands were fixed in 2% PFA for 20 min and processed for immunofluorescence, as previously described [[21](#_ENREF_21),[39](#_ENREF_39)]. In brief, Caco-2 cells and organoids in 3D culture were fixed in 2% PFA for 20 min at room temperature, washed in PBS, and permeabilized for 10 min in 0.5% Triton X-100 in PBS. Primary antibodies were diluted in block buffer and incubated overnight at 4°C. Cells were incubated with secondary antibodies and/or FITC-phalloidin for 1 h. DNA was stained and chamber slides were mounted using Vectashield mounting medium containing DAPI (Vector Scientific, Belfast, UK). Labelled cells were visualized using a Nikon 90i fluorescence microscope or a Leica SP5 laser scanning confocal microscope and images were processed using Elements (Nikon) or Leica LAS AF software. Sequential scan images were taken at the midsection of glands at room temperature using the Leica confocal on an HCX PL APO lambda blue 63 × 1.40 oil immersion objective at 1× or 2× zoom.

**Image processing**

Fluorescence microscopy images were processed using Leica Fw4000 Imaging software. Confocal images were processed, merged, and the mean area was quantified using LAS AF Leica Imaging Software.

**Analyses of cell nuclear morphology and DNA content with ImageJ**

In 3D organotypic and organoid cultures, spindle orientation, epithelial configuration, and lumen formation were assessed as previously defined [[21](#_ENREF_21),[31](#_ENREF_31)]. Spindle architecture was defined as bipolar or multipolar as outlined above. Bipolar spindle orientation was assessed by the angle between the spindle plane and a line drawn from the spindle midpoint to the gland centre using ImageJ, as previously described [[7](#_ENREF_7)]. Nuclear size, DNA content and ‘roundness’ were assessed in 3D glandular structures using ImageJ, as previously described [[70](#_ENREF_70)]. Roundness was computed using the border perimeter determined by the formula perimeter^2^/(4 × 𝜋× nuclear area) [[70](#_ENREF_70)].

**Human tumour samples and image analysis of immunohistochemistry data**

NHERF1 immunohistochemistry (IHC) was conducted in two CRC sample collections (A and B) provided by the Northern Ireland Biobank (NIB), while Aurora A immunofluorescence (IF) was assayed in sample A only. The present study involves samples relevant to biobank ORECNI application NIB13-0090, for which ethical approval is in place (ORECNI Ref No 11/NI/0013/-/NIB13-0090).

Sample A comprised 35 whole tumour sections and five matched normal mucosa specimens, while sample B was a tissue microarray (TMA) comprising 309 tumour CRC specimen cores across two TMA sections derived from 92 CRCs, as previously described [[31](#_ENREF_31)]. Sample B cores from rectal tumours that had received neoadjuvant radiotherapy (*n* = 28 cores) were excluded from analyses. NHERF1 IHC assays were conducted in the Northern Ireland Molecular Pathology Laboratory. Sections were cut at 4 μm thickness on a rotary microtome, dried overnight, and then stained in an automated immunostainer in accordance with the manufacturer’s instructions (Leica Bond-Max, Milton Keynes, UK). Sections were incubated with mouse monoclonal anti-NHERF-1 antibody (Lifespan Biosciences, Seattle, USA; LS-B1873; 1/200) after pretreatment with Bond-Max epitope retrieval solution 2 for 20 min. Primary antibody binding was detected using a polymer-based detection system (Bond Polymer Refine Detection, Newcastle Upon Tyne, UK; catalogue No DS9800) containing a peroxide block, post-primary polymer reagent 3,3′-diaminobenzidine tetrahydrochloride (DAB) chromogen solution, and haematoxylin counterstain. Stained sections were mounted in DPX. Apical localization of NHERF1 was defined as enrichment along the apical membrane domain of glandular structures [[78](#_ENREF_78)].

*Sample A*

Whole tumour/tissue sections were scored for apical NHERF1 intensity from 0 to 3, representing 0 = absent, 1 = weak, 2 = moderate, and 3 = strong apical staining. Scoring was conducted at 10× magnification over 40 fields per tumour/tissue section by two independent assessors. The highest intensity staining present in each field and the percentage of the field demonstrating this staining intensity within 20% increments were multiplied to provide a final score. A mean score per tumour section was derived from the average apical staining score across the 40 fields.

*Sample B*

Because of the small size of sample A, we conducted additional NHERF1 IHC assays in both cytoplasm and at the apical domain in TMA samples, prepared as outlined above. NHERF1 localization at apical regions of discernible glandular structures was scored 0–2, where 0 = absent, 1= moderate, and 2 = strong. Cytoplasmic staining was scored 0–3 as outlined above for sample A. Scores were multiplied by the percentage tumour cells with the particular staining intensity. Scoring was conducted by two independent assessors (JM and ML). Any discordant scores were resolved by consensus decision. As previously reported, PKCz p-T560 IHC was unsuccessful in formalin-fixed tissue sections [[21](#_ENREF_21)].

*Aurora A immunofluorescence*

Aurora A IF was conducted in sample A tumours only. Freshly cut 6 µm sections of tumour tissue (*n* = 34) or normal mucosa (*n* = 5) were used in assays of mitotic spindle architecture by Aurora A IF, as previously described [[46](#_ENREF_46)]. Stained sections were examined using a Nikon Eclipse Ti inverted two-channel fluorescence microscope (Nikon UK, Ltd, Surrey, UK). Sections were screened using a 63/1.3 oil objective lens. In mitotic cells, normal bipolar mitotic spindle architecture was characterized by two distinct polar Aurora A-positive signals, while multipolar mitoses were defined as more than two Aurora A-positive signals [[46](#_ENREF_46)]. Aurora A localizes to the centrosome during late S-phase at the time of centrosome replication [[79](#_ENREF_79)]. Cells with dispersed Aurora A were thought to represent early S-phase before re-localization to the centrosome, and those with only one Aurora A signal were thought to contain unseparated centrosomes during interphase. Both of these categories were excluded from the analysis. A total of 84.7 ± 4.7 high-power field images containing 180 ± 72 mitotic cells per section were captured as TIFF files using a Nikon Elements Imaging Software Viewer (Nikon, version 4.20) for review and analysis. By the above methods, each CRC specimen had a mean quantitative score for apical NHERF1 intensity and a mean value for multipolar spindle frequency, as a percentage of the total mitotic figures. We conducted correlation studies between these two quantitative endpoints.

*Clinicopathological demographics*

Clinicopathological data relevant to samples A and B held in the NIB were collected from Belfast Health and Social Services Trust Labcentre. The NIB has in place regulatory approvals for the NIB tumour collection to cover the use of archived tissues held in the cellular/tissue pathology laboratories of the NI HSC Trusts for appropriate research studies (REC reference 11/NI/0013), in accordance with the position statement issued in July 2009 by the Human Tissue Authority (HTA). In the present study, data were alphanumerically coded and linked with de-identified clinical and pathological information. No patient-identifiable information is released by the NIB to researchers.

**Data analysis**

Descriptive statistics were expressed as the mean ± SEM. Apical NHERF1 IHC data were skewed and were log-transformed to provide a normal distribution. Statistical analyses were by one- or two-way ANOVA, Student’s *t*-test or Pearson’s test of correlation using SPSS for Windows release 24.0 (IBM Corp, NY, USA) or GraphPad Prism software (v4.02; GraphPad Software Inc, La Jolla, CA, USA). Scatterplots, bar charts, and boxplots were used for display of quantitative numerical or categorical data.
