# Supplementary material for: Protein kinase C zeta suppresses low‐ or high‐grade colorectal cancer (CRC) phenotypes by interphase centrosome anchoring
Source: J Pathol. 2018 Mar 9;244(4):445–59. doi: 10.1002/path.5035 (PMC5873423; doi:10.1002/path.5035)
Supplement: Supplementary file 2 — Supplementary figure legends [file PATH-244-445-s009.docx]

**Supplementary figure legends**

**Figure S1 Dynamics of ezrin cap formation** **(supplementary)**

**A** Quantification of total ezrin/NHERF1 binding shown in **Figure 1A** after PKCz siRNA KD normalised to control nontargeting (NT) siRNA transfections = 0.66 ± 0.038; *p = 0.0123. **B** Immunoblots in Caco-2 cells after PKCz pseudosubstrate inhibitor (PKCzI) 1µM [[21](#_ENREF_21)] treatment or PKCz siRNA transfection versus vehicle only or nontargeting siRNA controls. Quantification of ezrin p-T567 ADU in Western blots shown in **Figure S1B** after PKCzI treatment **(C)** = 0.45 ± 0.05;**p = 0.009 or PKCz siRNA KD **(D)** = 0.54 ± 0.04; **p = 0.009. Values are normalised to control.

**E** Schematic of ezrin cap formation in relation to interphase centrosome anchoring, replication and clustering. Ezrin (red) is recruited from the cytosol to the cortex, where it becomes progressively restricted to form the ezrin cap, close to the interphase centrosome. The ezrin cap binds centrosomal astral microtubules (green) [[10](#_ENREF_10)]. Thus anchored to the cell cortex, the centrosome (orange) then replicates to generate one mother and one daughter centrosome (single curved arrow). In cancer, oncogenic processes drive abnormal centrosome replication (double curved arrow) to generate extra centrosomes (encircled) [[14](#_ENREF_14), [15](#_ENREF_15)]. Extra interphase centrosomes are thus clustered at the ezrin cap [[10](#_ENREF_10)]. **F** Cortical recruitment and restriction of ezrin p-T567 **F(i)** and total ezrin **F(ii)** in Caco-2 cells. Intervals of 3.5 h and 14 h after plating were suitable for assay of ezrin cortical recruitment and cap formation respectively. **G** Effects of PKCz siRNA KD on ezrin p-T567 cortical recruitment at

3.5 h shown in **Figure 1B,** *p= 0.012. **H** Effects of PKCz siRNA KD on NHERF1 cortical recruitment at 3.5 h shown in **Figure 1C,** **p=0.001. Values represent % cells with ezrin or NHERF1 cortical recruitment normalized against control. **I** Merlin cortical localization in control or PKCzI treated Caco-2 cells at 14 h after plating.

**J** Effects of Ez/Nhe pbi versus scrambled peptide control on total ezrin/NHERF1 binding shown in **Figure** **1D[i];** *p=0.012. **K** Effects of inhibitory peptide treatment on ezrin p-T567 cortical recruitment at 3.5 h after plating shown in **Figure 1D [ii],** **p=0.0035). **L** Effects of NHERF1 siRNA KD versus NT siRNA on NHERF1 expression. **M** Confocal assays of ezrin and NHERF1 localization at 14 h after plating in Caco-2 cells. NHERF1 does not localize at a cap. **N** Effects of PKCz siRNA KD (left panels) or Ez/Nhe pbi treatment (right panels) on ezrin cap formation in Caco-2 cells at 14 h after plating. All analyses by paired Student’s t test. Staining - DAPI (blue), ezrin p-T567 (red), merlin (red), total ezrin (green). NHERF1 (green). Scale bars = 20 μm.

**Figure S2 Summary effects of ezrin/NHERF1 interaction on multicellular morphogenesis**

**A** Summary of single lumen formation in control versus Ez/Nhe pbi-treated organoids shown in **Figure 2A**;*p=0.03 (n= 30 organoids per experimental condition in triplicate, expressed as %). **B** Nuclear “roundness” scores in control versus Ez/Nhe pbi treated organoids (measured roundness units [MRU]); *p= 0.02. **C** Nuclear area in control and Ez/Nhe pbi treated organoids shown in **Figure 2A,** p=NS. **D** Summary single lumen formation in control versus Ez/Nhe pbi-treated Caco-2 glands shown in **Figure 2C,****p=0.004. (n= 30 Caco-2 glands per experimental condition in triplicate, expressed as %). **E** Nuclear “roundness” scores in control versus Ez/Nhe pbi treated Caco-2 glands shown in **Figure 2C,** ***p <0.001. **F** Nuclear area in control and Ez/Nhe pbi treated Caco-2 cultures shown in **Figure 2C,** *p=0.012; n = 100 cells per experimental condition in triplicate. All analyses by paired Student’s t test.

**Figure S3 Summary effects of PKCz on mitotic spindle architecture in cells with extra centrosomes**

**A** Summary effects of PKCz siRNA KD versus control on centrosome clustering in Caco-2 cells shown in **Figure 3A** (right panels), **p = 0.001. **B** Centrosome clustering (insert) in PKCzI-treated U2OS cells versus control. **C** Summary effects of PKCzI versus control on centrosome clustering in U2OS cells shown in **B,** *p = 0.05. **D** Summary effects of NHERF siRNA KD on centrosome clustering in Caco-2 cells shown in **Figure 3C**, *p = 0.03. **E** Centrosome clustering (insert) in NHERF1 siRNA transfected B549 cells versus control. **F** Summary effects of NHERF1 siRNA KD versus control on centrosome clustering in B549 cells shown in **E**;** p = 0.005.

**G** Mitotic spindle architecture in Ez/Nhe pbi-treated Caco-2 cells versus control. **H.** Summary effects of Ez/Nhe pbi-treatment versus control on mitotic spindle architecture in Caco-2 cells shown in **G**; bipolar - *p=0.01; Multipolar - **p <0.001. Clustering was assessed in n=100 cells with > 2 centrosomes in triplicate. Spindle architecture was assessed in 100 mitotic cells in triplicate, expressed as a percentage. **I** Doxocycline-inducible PLK4 overexpression (PLK4OE) in Caco-2 and HCT116 cells. **J** Summary of doxycycline-inducible PLK4 ADU in Caco-2 and HCT116 cells shown in **I**. **K** Centrosome amplification in PLK4 overexpressing (PLK4OE) Caco-2 and HCT116 cells versus control. Assays at 24h after doxycycline-driven PLK4 overexpression. **L** Summary effects of PLK4OE on centrosome number in Caco-2 **p = 0.006 and HCT116 cells **p=0.007, shown in **K**.

Analyses by paired Student’s t test (A, C, D, F, L) or ANOVA (H). Staining - DAPI (blue), pericentrin (red) and α-tubulin (green).

**Figure S4 Effectiveness of siRNA PKCz knockdown.**

**A** PKCz expression after siRNA knockdown versus control in Caco-2 PLK4OE cells.

**B** Summary effects of PKCz siRNA KD versus control on PKCz ADU in Caco-2 PLK4OE cells shown in **A,**** p=0.008; paired Student’s t test.

**Figure S5 Spindle architecture in control versus Ez/Nhe pbi-treated Caco-2 cultures and associated quantitative data.**

**A** Spindle architecture (inserts) in control versus Ez/Nhe pbi-treated Caco-2 cultures. **B** Summary effects of Ez/Nhe pbi treatment versus control on spindle architecture shown in **A**; bipolar - **p =0.008; Multipolar - **p = 0.003. (100 mitotic cells assessed in triplicate in each experimental condition). Analysis by ANOVA. Staining DAPI (blue), pericentrin (red), α-tubulin (green).

**Figure S6 Graphic Summary - Effects of defective centrosome anchoring on evolution of CRC morphological and/or genomic phenotypes.**

1. Ezrin cap (red) anchoring of the interphase centrosome through astral microtubule binding (green). Thus stabilized, the centrosome replicates to generate 2 normal or extra (encircled) centrosomes [[10](#_ENREF_10)]. **(ia1 and ia2)** show bipolar spindle assembly and normal orientation with normal **(ia1)** or clustered **(ia2)** centrosomes. **(ib)** shows correct bipolar spindle orientation, normal cleavage furrows and appropriate apical membrane (AM; red) alignment **(ic)** shows lumen expansion driven by secretion (blue arrows). These steps enable appropriate multicellular assembly and gland formation [[7](#_ENREF_7)]. **(id-ie)** show representative images in the culture model and normal colon.
2. Defective ezrin cap formation with a single interphase centrosome. Impaired anchoring of the interphase centrosome to the cell cortex drives bipolar spindle misorientation **(iia)** [[55](#_ENREF_55)]. In turn, bipolar spindle misorientation drives apical membrane AM (red) misalignment and aberrant planes of cell cleavage [[7](#_ENREF_7)] **(iib).** Collectively, these processes induce cribriform multicellular morphology **(iic)** [[31](#_ENREF_31)]. (**iid-iie)** show representative images in the culture model and low-grade colorectal cancer
3. Defective ezrin cap formation with extra centrosomes. Dispersal of multiple, unanchored centrosomes promotes transient multipolar spindle formation [[16](#_ENREF_16),[42](#_ENREF_42)]. **(iiia)** Thus formed, most transient multipolar spindles are converted to misorientated, pseudobipolar spindles by error-prone metaphase clustering mechanisms, accompanied by chromosome lag (shown in the cartoon) [[16](#_ENREF_16)] **(iiib1)**. A few cells with multipolar spindles undergo multipolar division to generate pleomorphic progeny [[16](#_ENREF_16)] **(iiib2).** Segregation error associated with these processes promotes chromosomal instability (CIN). In the present study, we show whole chromosome (Chr) aneuploidy indicated by 3 x Chr1 (green) signals **(iiic1).** CIN arising from these mechanisms is accompanied by nuclear pleomorphism, gross multicellular perturbation and extrusion of genomically unstable cells across basal interface with ECM, shown in cartoon **(iiic2)** and culture model **(iiid).** Insets show bipolar or multipolar spindle architecture. Extrusion of malignant cells from main epithelial mass in high grade CRC shown in **(iiie).**
