# Supplementary material for: Protein kinase C zeta suppresses low‐ or high‐grade colorectal cancer (CRC) phenotypes by interphase centrosome anchoring
Source: J Pathol. 2018 Mar 9;244(4):445–59. doi: 10.1002/path.5035 (PMC5873423; doi:10.1002/path.5035)
Supplement: Supplementary file 9 — Table S1. Antibodies, suppliers, catalogue numbers, and dilutions used [file PATH-244-445-s003.doc]

**Table S1.** Antibodies, suppliers, catalogue numbers, and dilutions used

| **Antibody** | **Supplier** | **Catalogue ID** | **Dilution for western** | **Dilution for confocal/IHC/IF** |
| --- | --- | --- | --- | --- |
| PKCz | Abcam | Ab57432 | 1/1000 | NA |
| p-PKCz | Abcam | Ab59412 | 1/500 | 1/200 |
| PLK4 | Abcam | Ab71394 | 1/1000 | NA |
| Ezrin | Abcam | Ab4069 | 1/1000 | 1/200 |
| p-Ezrin | Abcam | Ab47293 | 1/500 | 1/100 |
| NHERF1 | Lifespan Biosciences | LS-C44851-100 | 1/500 | NA |
| NHERF1 | Lifespan Biosciences | LS-B1873 | NA | 1/200 |
| Pericentrin | Abcam | Ab4448 | NA | 1/200 |
| Alpha-tubulin | Abcam | Ab4074 | NA | 1/200 |
| Merlin | Santa Cruz Biotechnology | SC332 | NA | 1/200 |
| GAPDH | Abcam | Ab8245 | 1/5000 | NA |
| Aurora A (phospho T288) | BD Transduction | Clone 4 IAK1 | NA | 1/200 |
